# Supplemented Low-Protein Diet May Delay the Need for Preemptive Kidney Transplantation: A Nationwide Population-Based Cohort Study

**DOI:** 10.3390/nu13093002

**Published:** 2021-08-28

**Authors:** Chieh-Li Yen, Pei-Chun Fan, George Kuo, Chao-Yu Chen, Ya-Lien Cheng, Hsiang-Hao Hsu, Ya-Chun Tian, Antoine Chatrenet, Giorgina Barbara Piccoli, Chih-Hsiang Chang

**Affiliations:** 1 Kidney Research Center, Department of Nephrology, Chang Gung Memorial Hospital, Linkou Branch, Taoyuan 33305, Taiwan; b9102087@yahoo.com.tw (C.-L.Y.); franwis1023@gmail.com (P.-C.F.); b92401107@gmail.com (G.K.); Chaoyuclaire@gmail.com (C.-Y.C.); yolien0205@gmail.com (Y.-L.C.); hsianghao@gmail.com (H.-H.H.); dryctian@cgmh.org.tw (Y.-C.T.); 2 Néphrologie, Centre Hospitalier du Mans, 72037 Le Mans, France; antoine.chatrenet@gmail.com (A.C.); gbpiccoli@yahoo.it (G.B.P.); 3 Dipartimento di Scienze Cliniche e Biologiche, Universitàdi Torino, 10100 Torino, Italy

**Keywords:** chronic kidney disease, low protein diet, amino acid and keto acid supplementation, preemptive kidney transplantation, adverse events

## Abstract

Background: Although several studies suggest the benefit of a low-protein diet supplemented with amino acids and keto acids (sLPD) in delaying the initiation of hemodialysis, evidence on whether these nutritional approaches could delay the timing of preemptive transplantation is lacking. Methods: Retrospective nationwide cohort study, from Taiwan’s National Health Insurance Research Database. Patients having undergone a first preemptive kidney transplantation between 2001 and 2017 were identified and divided into two groups according to the presence of sLPD treatment or not. The primary outcome was the time between the diagnosis of advanced CKD and transplantation. Secondary outcomes were post-transplantation adverse events. Results: A total of 245 patients who received their first preemptive kidney transplantation were identified from the nationwide database; 63 of them had been on an sLPD prior to transplantation (sLPD group). The duration between the day of advanced CKD diagnosis and the day of transplantation was significantly longer in the sLPD group compared with the non-sLPD group (median duration: 345 vs. 220 days, *p* = 0.001). The risk of post-transplantation adverse events did not differ between the two groups. Conclusions: Within the limits of its observational, retrospective design, this is the first study to suggest that nutritional management with sLPDs can safely delay the timing of preemptive kidney transplantation.

## 1. Introduction

Uremia has been classically defined as a state of protein intoxication, and protein metabolism plays a major role in several pathophysiological alternations in advanced chronic kidney disease (CKD) [1,2,3,4]. Therefore, protein restriction has been considered a treatment option for advanced CKD for more than a century [5,6].

In the 1970s, Walser et al. first reported that a very low-protein diet supplemented with ketoanalogues of essential amino acids can safely delay the progression of CKD [7]. An added advantage of ketoanalogues is that they can be converted into essential amino acids by transamination, thus reducing nitrogenous waste products, such as urea [8]. This transamination effect is effective if the daily protein intake is reduced [9]. Indeed, several randomized control trials [10,11,12] and observational cohort studies [13,14,15] have demonstrated the benefit of supplemented diets, with different protein contents (0.6 g/Kg/day in the sLPDs and 0.3–0.4 g/kg/day in the “very low” supplemented diet, or sVLPD), in slowing the progression of CKD, and in delaying the need to start dialysis. More importantly, these studies have demonstrated that well prescribed, and adequately controlled, sLPDs and sVLPDs do not increase the risk of protein-energy wasting, nor the risk of death [16,17,18,19].

Furthermore, large observational studies have reported that patients on dietary treatment had a lower risk of death or infection after commencing dialysis [13,20]. Most of these studies were performed in settings in which dialysis was available without restrictions, and in which the current end points of death or start of dialysis usually occurred in populations of a high mean age.

Although there is growing evidence of the effect of sLPDs and sVLPDs in delaying the need to start dialysis, the issue regarding the need for preemptive kidney transplantation has not been fully explored. Indeed, preemptive kidney transplantation is a choice that may be of particular relevance to young patients, and in settings in which dialysis availability is restricted. To the best of our knowledge, only one paper has addressed this issue, reporting on a small series of cases successfully managed on a moderately restricted supplemented diet while waiting for preemptive pancreas-kidney transplantation [21]. Delaying the need for kidney transplantation is important for several reasons: first, the lifespan of an allograft is limited. For example, in the United States, the 5-year allograft survival rate is approximately 85% for living donors and 72% for deceased donors [22]. Second, the exposure to immunodepressive drugs is not without side effects, which are cumulative over time. Delaying the need for such exposure, and associated risks of developing infections or cancer, may be an advantage. Third, immunosuppressive treatments are improving over time, and delaying the need for transplantation may mean having access to better drugs, or drug combinations [23]. Thus, this study aimed to elucidate the effect of sLPDs in delaying the need for preemptive kidney transplantation, and to evaluate if such diets could have a detrimental long-term influence after kidney transplantation.

Taking advantage of the comprehensive nationwide medical records collected in Taiwan by the National Health Insurance Research Database (NHIRD), this study investigated the association between the use of sLPDs and the time to surgery among patients who received a preemptive kidney transplantation. In addition, this study aimed to check for eventual adverse effects of sLPDs on survival, and on the risk of major complications after transplantation.

The novelty of the study, aimed at trying to answer an unanswered question, resides in the study population, relatively young and with low comorbidity, since the characteristics of the patients receiving kidney transplantations differed from those of the usual CKD patients receiving dialysis, in terms of age and comorbidity burden [24,25,26].

## 2. Materials and Methods

### 2.1. Data Source

This retrospective cohort study utilized the National Health Insurance (NHI) Research Database (NHIRD) collected by the National Health Informatics Project (NHIP) and managed by the Health and Welfare Data Science Center (HWDC) of Taiwan. In March 1995, Taiwan launched a nationwide, single-payer, compulsory health care program called the NHI program, which covers approximately 99.8% of Taiwan’s population (nearly 24 million people in 2017). Comprehensive health care information on insured patients, including outpatient visits, disease diagnoses, drug prescriptions, and procedures, is included in the NHIRD. Disease diagnoses in the NHIRD follow the International Classification of Diseases, 9th Revision, Clinical Modification (ICD-9-CM) before 2015, and ICD-10-CM after 2016. Although examination reports and laboratory data are not available, the population-wide comprehensiveness is the strength of the database. Further information regarding the NHI and NHIRD is available in previous publications [27,28,29]. The current study based on the NHIRD qualified for a waiver of consent from the Chang Gung Medical Foundation’s Institutional Review Board (approval number: 201800002B0).

### 2.2. Patient Selection

**Figure 1:**
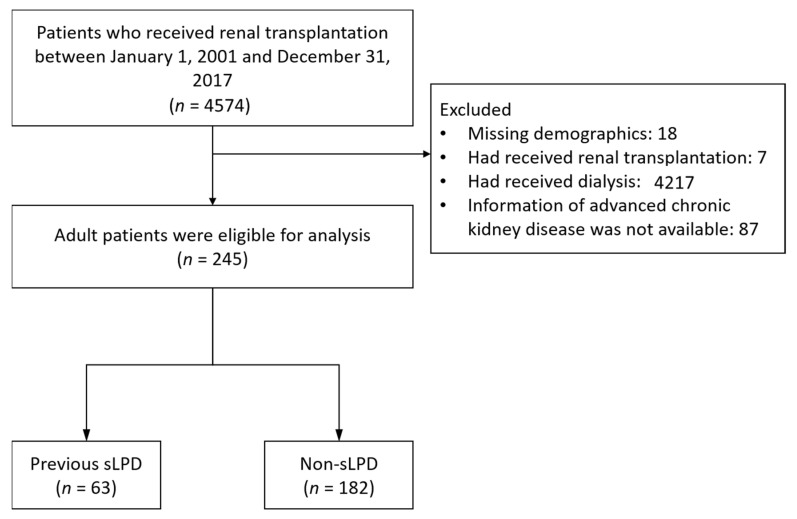
Inclusion criteria of the study patients. sLPD: ketoanalogue-supplemented low-protein diet.

Patients who received their first kidney transplantation between 2001 and 2017 were identified from the NHIRD, covering the entire Taiwanese population. The diagnosis code 55.69 (ICD-9-CM), Z94.0 (ICD-10-CM), the procedure code V42.0 (ICD-9-CM), 0TY00Z0, 0TY00Z2 (ICD-10-PCS) and the Taiwan reimbursement code 76020 were used in combination to identify kidney transplantation according to hospitalization data of NHIRD. The discharge date after hospitalization for kidney transplantation was defined as the index date. Patients who had a history of kidney transplantation or had received dialysis before the index date were excluded. Finally, patients whose information of advanced CKD was not available were also excluded (Figure 1).

The kidney transplant donors were not identifiable from the NHIRD. Taiwan has implemented very strict regulations on organ donations, banning organ trafficking and trading; thus, the process of transplantation in Taiwan takes place without the exchange of money. In this study, patients receiving kidney transplantations were identified from the Taiwan’s NHIRD, which indicated that they all received their living donor kidney transplantations in Taiwan and followed the strict regulations.

### 2.3. Exposure

Patients with preemptive kidney transplantation were divided into two groups, depending on the use of ketoanalogue supplementation between the date of diagnosis of advanced CKD, and the date of kidney transplantation. The date of diagnosis of advanced CKD was defined as the date of first use of erythropoiesis- stimulating agents (ESAs) after at least three outpatient diagnoses of CKD. In accordance with the NHI reimbursement regulations, only patients with serum creatinine >6 mg/dL in combination with a hematocrit <28% qualify for ESA treatment. Therefore, this has been a commonly used method to identify patients with CKD stage 5 in previous NHIRD studies [30,31]. Similarly, ketoanalogue supplementation with a maximum dose of six tablets of ketosteril per day can only be prescribed without copayment to patients whose serum creatinine exceeds 6 mg/dL and who can comply with an LPD. In addition, according to the regulations of Taiwan’s Food and Drug Administration, Ketosteril can only be prescribed by doctors and is not allowed to be purchased or collected without prescription. Thus, the users of Ketosteril can be identified by using NHIRD. These patients receive regular nutritional checks and their diets are usually plant-based and moderately to severely protein-restricted. Therefore, according to NHI, the use of the drug Ketosteril is a surrogate for demonstrating a concomitant sLPD.

### 2.4. Covariates

The covariates analyzed were age, sex, primary kidney disease, comorbid conditions (hypertension, diabetes mellitus, dyslipidemia, gouty arthritis, peptic ulcer/gastroesophageal reflux disease, ischemic heart disease, and liver cirrhosis), the Charlson Comorbidity Index (CCI), a history of hospitalization for events (heart failure, ischemic stroke, hemorrhagic stroke and myocardial infarction), and medications, namely anti-platelet agents, angiotensin converting enzyme inhibitors (ACEi), angiotensin receptor blockers (ARB), non-steroidal anti-inflammatory drugs (NSAID), statins, iron, vitamin D, calcium, oral hypoglycemic agents (OHA), and insulin. The diagnosis of comorbidities was defined as having at least two outpatient diagnoses or one inpatient diagnosis in the year preceding the index date. The history of hospitalization was traced back to 1995. Most of the diagnostic codes used in this study have been validated in previous NHIRD studies [32,33]. Information on medications was extracted 3 months before and after the index date, and was extracted from the claims data of outpatient visits, or the refill prescriptions for chronic illness in the pharmacy using the Anatomical Therapeutic Chemical codes, or Taiwan NHI reimbursement codes. The ICD diagnostic codes used in this study are provided in the supplement (Supplemental Table S1).

### 2.5. Outcomes

The main outcome was the duration between the date of advanced CKD diagnosis and the date of receiving kidney transplantation. The secondary outcomes were adverse events after kidney transplantation, including all-cause mortality, infection-associated hospitalization or death, newly diagnosed malignancy, osteoporosis-related fracture, new-onset diabetes mellitus, allograft failure requiring maintenance dialysis, and major cardiac and cerebrovascular events (MACCE). The latter were composed of acute myocardial infarction, acute ischemic stroke, intracerebral hemorrhage, heart failure hospitalization, and cardiovascular death. Information of date, place, and causes of mortality were available in the Taiwan Death Registry database which were included in HWDC. Components of MACCE were detected using a principal discharge diagnosis. Infection-related hospitalization, including sepsis, was detected using the principal or secondary discharge diagnoses. Osteoporosis-related fracture was identified on the basis of the principal diagnosis of a hospitalization or emergency visit. Newly diagnosed malignancy and allograft failure requiring maintenance dialysis were identified from the catastrophic illness certificate sub-database of the NHIRD. New-onset diabetes mellitus required at least two newly performed outpatient diagnoses with a prescription of oral hypoglycemic agents (including sulfonylurea, metformin, alpha-glucosidase inhibitors, thiazolidinediones, meglitinides, dipeptidyl peptidase-4 inhibitor, glucagon-like peptide 1 receptor agonist and sodium-glucose cotransporters inhibitor) or insulin.

### 2.6. Statistical Analysis

The mean duration from the day of advanced CKD to the day of transplantation was compared using the Mann–Whitney U-test between the sLPD and non-sLPD groups. The risk of adverse events (MACCE) or death (all-cause mortality, cardiovascular death, death from infection or sepsis) after transplantation was compared using a Cox proportional hazard model. The risk of non-fatal outcomes (i.e., new-onset diabetes) after transplantation was compared using the Fine and Gray subdistribution hazard model which considered death during follow-up as a competing risk. To control for confounders, we created a propensity score in which the study group (sLPD vs. non-sLPD) was regressed on the patient’s characteristics, and we additionally adjusted for the propensity score in the survival analyses [34]. Variables included in the propensity score calculation are listed in Table 1 along with the index date. A two-sided *p* value < 0.05 was considered statistically significant. All statistical analyses were performed using SAS version 9.4 (SAS Institute, Cary, NC, USA).

## 3. Results

### 3.1. Trend of Preemptive and Total Kidney Transplantations in Taiwan

**Figure 2:**
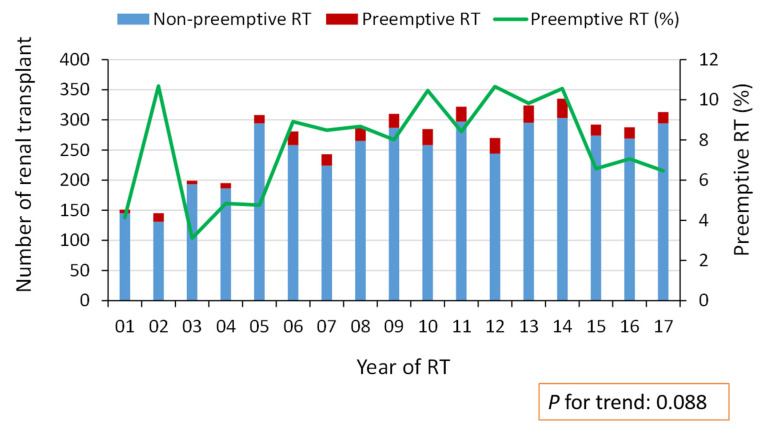
Number of annual preemptive and total renal transplantations in Taiwan from 2001 to 2017. RT: renal transplantation.

Figure 2 displays the annual number of preemptive and total kidney transplantations in Taiwan according to the NHIRD between 2001 and 2017. The annual number of kidney transplantations increased from 151 patients in 2001 to around 300 patients after 2013. On the other hand, the proportion of preemptive kidney transplantations also gradually increased from 4.1% (6/151) of patients in 2001 to a peak of 10.7% (26/270) in 2012. However, the proportion of preemptive kidney transplantations dropped after 2015.

### 3.2. Patient Characteristics

A total of 245 patients who received their first preemptive kidney transplantation between 2001 and 2017 were identified from the NHIRD. Among them, 63 had been on a supplemented low protein diet (sLPD group), whereas the remaining 182 patients had not (non-sLPD group). Table 1 displays the baseline clinical characteristics of the patients. Patients on sLPD were significantly older than those without (47.5 vs. 42.5 years; *p* = 0.022). Patients in the sLPD group were more likely to be male (58.7% vs. 44.5%), though this was not significant (*p* = 0.052). Except for age, there were no significant differences of the baseline characteristics between groups.

### 3.3. Outcomes

**Figure 3:**
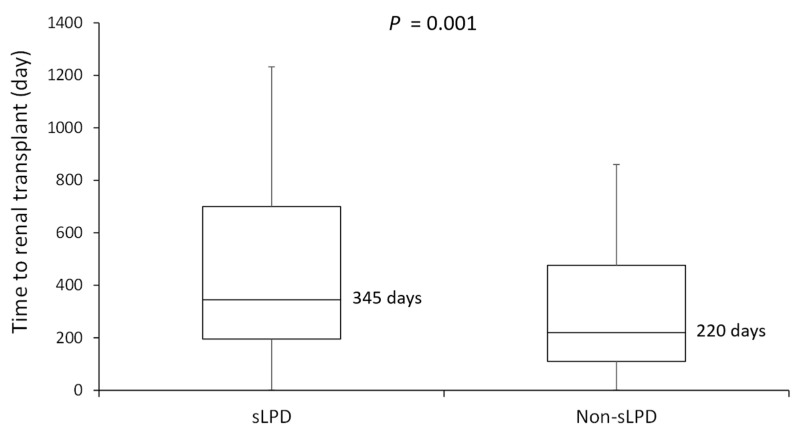
Median duration from the day of advanced CKD diagnosis to the day of kidney transplantation in the sLPD and non-sLPD groups. sLPD, ketoanalogue-supplemented low-protein diet.

As illustrated in Figure 3, the sLPD group showed a significantly longer duration between the day of advanced CKD diagnosis and the day of kidney transplantation than the non-sLPD group (*p* = 0.001). The median duration was 345 days (interquartile range: 195–700 days) in the sLPD group and 220 days in the non-sLPD group (interquartile range: 110–476 days), respectively.

The mean follow-up duration after kidney transplantation was 6.7 years (standard deviation: 4.1 years) among the 245 patients. No significant difference between groups was found in terms of all-cause mortality, MACCE, infection-related hospitalization or death, newly diagnosed malignancy, new-onset diabetes mellitus, osteoporosis-related fracture, or allograft failure requiring dialysis (Table 2).

## 4. Discussion

The importance of supplemented low-protein diets has been recently underlined in the new KDOQI guidelines, principally for their advantage of allowing a longer follow-up in the pre-dialysis phase [35]. High-quality diets with appropriate restriction of protein have been regarded as a crucial treatment in CKD population [36]. However, to the best of our knowledge, only one small study had focused on the advantage of this policy while waiting for pancreas-kidney transplantation, and no study had been performed to analyze the effect of this dietary approach on delaying time to transplantation in patients scheduled for living-donor kidney transplantation [21].

Our study was performed in Taiwan, employing the NHIRD, a comprehensive nationwide database; it suggests that sLPDs may safely delay the timing of preemptive kidney transplantation among patients with advanced CKD.

In Taiwan, patients who receive preemptive kidney transplantations account for a very small number of patients with ESRD. Only 245 recipients between 2001 and 2017 were identified. Even in Western countries, where organ donation is more popular than in Asian countries, preemptive kidney transplantation remains a niche [37,38].

Within the limits of a small number of cases, our study suggests that an sLPD treatment can significantly delay the timing of preemptive kidney transplantation, by a median of 125 days. Even more importantly, this study suggests that the most severe posttransplant adverse events, namely all-cause mortality, cardiovascular diseases, allograft failure requiring dialysis, new-onset diabetes mellitus, fracture, and malignancy, were not increased in patients who had been on a supplemented diet before kidney transplantation.

Another point of interest is related to the characteristics of the study group: the mean age of our patients was 43.8 years, remarkably lower than the age of patients starting chronic dialysis (presently around 61 years), as reported in previous NHIRD-based studies [14,20]. Furthermore, patients in this study had fewer comorbidities compared with the overall patients with advanced CKD [31,39]. For example, in the study group, only 15.5% of patients had diabetes mellitus, and the median Charlson index was only 2 (see above), mainly dependent on severe CKD. In this regard, this study supports the interest of offering a supplemented LPD to young CKD patients.

Several limitations of the current study should be acknowledged. First, the prevalence of preemptive kidney transplantations in Taiwan is lower than the global average. Therefore, even after using a nationwide database, the number of enrolled patients was limited in this study. Second, laboratory data, including serum albumin, creatinine, lipid profile, proteinuria, and hemoglobin, were not available in the NHIRD. In addition, the information on medical institutions and socioeconomic status of enrollees was not available in the dataset, and differences among these covariates may theoretically influence the results. Third, in an observational study, removing all potential confounders is impossible regardless of the study design, which may further bias the results. Namely, the possibility cannot be excluded that patients with a slower progression of CKD, or with better compliance, were selected. Fourth, this study was based on a Taiwanese database. Therefore, the results of the current study may not apply entirely to other populations because of cultural, dietary, and genetic differences. Fourth, although this study suggests that the use of sLPDs may delay the need of preemptive kidney transplantation, and without increasing short-term adverse events, it was not clear if delaying access to the transplantation would result in longer life expectancy or better quality of life. Lastly, the details of the diets were not available in the database, and the characteristics of the donors could not be identified.

Most of these limitations are linked to the study design; however, a randomized control study would be hardly feasible, as well as possibly unsound from an ethical point of view, since diets need commitment and compliance is crucial [40,41].

Accepting these limitations, in conclusion, this study suggests that sLPDs have the potential to delay the timing of preemptive kidney transplantation, without increasing the risk of post-transplantation adverse events. Further well-designed randomized and prospective studies are warranted to verify these findings.

## Figures and Tables

**Table 1:** Baseline characteristics of the patients who received preemptive kidney transplantation, by previous treatment with sLPD.

Variable	Total (*n* = 245)	sLPD (*n* = 63)	Non-sLPD (*n* = 182)	*p*
Age (years; mean ± SD)	43.8 ± 15.0	47.5 ± 13.4	42.5 ± 15.4	0.022
Male	118 (48.2)	37 (58.7)	81 (44.5)	0.052
Primary kidney disease				0.151
Interstitial nephritis	24 (9.8)	6 (9.5)	18 (9.9)	
Obstructive nephropathy	NA	NA	NA	
Polycystic kidney	NA	NA	NA	
Hypertension nephropathy	131 (53.5)	42 (66.7)	89 (48.9)	
Diabetes nephropathy	6 (2.5)	0 (0.0)	6 (3.3)	
Chronic glomerulonephritis	50 (20.4)	10 (15.9)	40 (22.0)	
Others	27 (11.0)	NA	NA	
Comorbid condition				
Hypertension	194 (79.2)	53 (84.1)	141 (77.5)	0.262
Diabetes mellitus	38 (15.5)	13 (20.6)	25 (13.7)	0.192
Dyslipidemia	60 (24.5)	17 (27.0)	43 (23.6)	0.593
Gouty arthritis	41 (16.7)	11 (17.5)	30 (16.5)	0.858
Peptic ulcer/GERD	25 (10.2)	6 (9.5)	19 (10.4)	0.836
Ischemic heart disease	14 (5.7)	4 (6.4)	10 (5.5)	0.801
Liver cirrhosis	3 (1.2)	0 (0.0)	3 (1.7)	0.305
CCI total score median [Q1–Q3]	2 (2–3)	2 (2–3)	2 (2–3)	0.837
CCI total score group				0.698
2	145 (59.2)	39 (61.9)	106 (58.2)	
3	50 (20.4)	11 (17.5)	39 (21.4)	
4	27 (11.0)	5 (7.9)	22 (12.1)	
5	9 (3.7)	3 (4.8)	6 (3.3)	
≥6	14 (5.7)	5 (7.9)	9 (5.0)	
History of event				
Heart failure hospitalization	6 (2.5)	NA	NA	0.608
Ischemic stroke	NA	NA	NA	0.430
Hemorrhage stroke	NA	NA	NA	0.556
Myocardial infarction	0 (0.0)	0 (0.0)	0 (0.0)	-
Medication				
Antiplatelet	19 (7.8)	7 (11.1)	12 (6.6)	0.248
ACEi/ARB	107 (43.7)	29 (46.0)	78 (42.9)	0.662
NSAID + COX-II inhibitors	3 (1.2)	0 (0.0)	3 (1.7)	0.305
Statin	67 (27.4)	21 (33.3)	46 (25.3)	0.216
Iron supplement	67 (27.4)	15 (23.8)	52 (28.6)	0.465
Vitamin D therapy	62 (25.3)	14 (22.2)	48 (26.4)	0.514
Calcium supplementation	112 (45.7)	26 (41.3)	86 (47.3)	0.411
OHA	38 (15.5)	11 (17.5)	27 (14.8)	0.620
Insulin	25 (10.2)	6 (9.5)	19 (10.4)	0.836

Abbreviations: sLPD, ketoanalogue-supplemented low-protein diet; SD, standard deviation; NA, not available due to the number of observations less than 3 not being allowed to be revealed according to the regulation of Health and Welfare Data Center; GERD, gastroesophageal reflux disease; CCI, Charlson Comorbidity Index; ACEi, angiotensin converting enzyme inhibitor; ARB, angiotensin receptor blocker; NSAID, non-steroidal anti-inflammatory drug; COX-II, cyclooxygenase II; OHA, oral hypoglycemic agent.

**Table 2:** Follow-up outcomes of patients after receiving preemptive kidney transplantation, by previous treatment sLPD.

Outcome	Total (*n* = 245)	sLPD (*n* = 63)	Non-sLPD (*n* = 182)	Unadjusted HR (95% CI)	Adjusted HR (95% CI) *
All-cause mortality	18 (7.4)	1 (1.6)	17 (9.3)	0.31 (0.04, 2.36)	0.42 (0.05, 3.67)
Cardiovascular event					
Acute myocardial infarction	NA	NA	NA	-	-
Acute ischemic stroke	NA	NA	NA	2.95 (0.46, 18.90)	1.95 (0.35, 10.95)
Intracerebral hemorrhage	NA	NA	NA	-	-
Heart failure hospitalization	NA	NA	NA	-	-
Cardiovascular death	5 (2.0)	0 (0.0)	5 (2.8)	-	-
Infection death	6 (2.5)	1 (1.6)	5 (2.8)	1.06 (0.12, 9.31)	0.87 (0.07, 10.44)
Sepsis-related hospitalization	19 (7.8)	3 (4.8)	16 (8.8)	0.87 (0.26, 2.95)	1.29 (0.28, 5.98)
Sepsis death	6 (2.5)	1 (1.6)	5 (2.8)	1.06 (0.12, 9.31)	0.87 (0.07, 10.44)
Newly diagnosed malignancy	22 (9.0)	5 (7.9)	17 (9.3)	1.74 (0.65, 4.64)	1.21 (0.42, 3.52)
Osteoporosis-related fracture	13 (5.3)	NA	NA	0.85 (0.19, 3.84)	1.18 (0.26, 5.36)
New-onset diabetes	24 (9.8)	6 (9.5)	18 (9.9)	1.26 (0.51, 3.13)	1.20 (0.39, 3.66)
Dialysis after transplant	32 (13.1)	5 (7.9)	27 (14.8)	0.99 (0.37, 2.65)	1.21 (0.40, 3.70)

Abbreviations: sLPD, ketoanalogue-supplemented low-protein diet; RT, kidney transplantation; SD, standard deviation; HR, hazard ratio; CI, confidence interval; *B*, regression coefficient; NA, not available due to a number of observations less than 3 not being allowed to be revealed, according to the regulation of Health and Welfare Data Center; * adjusted for propensity score.

## Data Availability

The data are not publicly available due to the regulations of NHIRD.

## Supplementary Materials

The following are available online at https://www.mdpi.com/article/10.3390/nu13093002/s1, Supplement Table S1. Provided ICD-9 CM diagnostic codes used in this study.

## References

[1] Fukagawa M., Drueke T.B. (2013). Introduction: Expanding concepts of chronic kidney disease-mineral and bone disorder (ckd-mbd). Kidney Int. Suppl..

[2] Akchurin O.M., Kaskel F. (2015). Update on inflammation in chronic kidney disease. Blood Purif..

[3] Tanaka H., Komaba H., Koizumi M., Kakuta T., Fukagawa M. (2012). Role of uremic toxins and oxidative stress in the development of chronic kidney disease-mineral and bone disorder. J. Ren. Nutr..

[4] Desjardins L., Liabeuf S., Oliveira R.B., Louvet L., Kamel S., Lemke H.D., Vanholder R., Choukroun G., Massy Z.A., European Uremic Toxin Work Group (2014). Uremic toxicity and sclerostin in chronic kidney disease patients. Nephrol. Ther..

[5] Lewis D.S. (1921). On the influence of a diet with high protein content on the kidney. Can. Med. Assoc. J..

[6] Addis T., Lew W. (1939). Diet and death in acute uremia. J. Clin. Investig..

[7] Walser M. (1975). Ketoacids in the treatment of uremia. Clin. Nephrol..

[8] Walser M., Lund P., Ruderman N.B., Coulter A.W. (1973). Synthesis of essential amino acids from their alpha-keto analogues by perfused rat liver and muscle. J. Clin. Investig..

[9] Kang C.W., Tungsanga K., Walser M. (1986). Effect of the level of dietary protein on the utilization of alpha-ketoisocaproate for protein synthesis. Am. J. Clin. Nutr..

[10] Walser M., Hill S., Ward L. (1992). Progression of chronic renal failure on substituting a ketoacid supplement for an amino acid supplement. J. Am. Soc. Nephrol..

[11] Walser M., Hill S.B., Ward L., Magder L. (1993). A crossover comparison of progression of chronic renal failure: Ketoacids versus amino acids. Kidney Int..

[12] Jiang N., Qian J., Sun W., Lin A., Cao L., Wang Q., Ni Z., Wan Y., Linholm B., Axelsson J. (2009). Better preservation of residual renal function in peritoneal dialysis patients treated with a low-protein diet supplemented with keto acids: A prospective, randomized trial. Nephrol. Dial. Transplant..

[13] Bellizzi V., Chiodini P., Cupisti A., Viola B.F., Pezzotta M., De Nicola L., Minutolo R., Barsotti G., Piccoli G.B., Di Iorio B. (2015). Very low-protein diet plus ketoacids in chronic kidney disease and risk of death during end-stage renal disease: A historical cohort controlled study. Nephrol. Dial. Transplant..

[14] Wu C.H., Yang Y.W., Hung S.C., Kuo K.L., Wu K.D., Wu V.C., Hsieh T.C., National Taiwan University Study Group on Acute Renal Failure (NSARF) (2017). Ketoanalogues supplementation decreases dialysis and mortality risk in patients with anemic advanced chronic kidney disease. PLoS ONE.

[15] Yen C.L., Fan P.C., Lee C.C., Kuo G., Tu K.H., Chen J.J., Lee T.H., Hsu H.H., Tian Y.C., Chang C.H. (2020). Advanced chronic kidney disease with low and very low gfr: Can a low-protein diet supplemented with ketoanalogues delay dialysis?. Nutrients.

[16] Walser M., Mitch W.E., Maroni B.J., Kopple J.D. (1999). Should protein intake be restricted in predialysis patients?. Kidney Int..

[17] Kopple J.D., Monteon F.J., Shaib J.K. (1986). Effect of energy intake on nitrogen metabolism in nondialyzed patients with chronic renal failure. Kidney Int..

[18] Piccoli G.B., Nazha M., Capizzi I., Vigotti F.N., Mongilardi E., Bilocati M., Avagnina P., Versino E. (2016). Patient survival and costs on moderately restricted low-protein diets in advanced ckd: Equivalent survival at lower costs?. Nutrients.

[19] Piccoli G.B., Ventrella F., Capizzi I., Vigotti F.N., Mongilardi E., Grassi G., Loi V., Cabiddu G., Avagnina P., Versino E. (2016). Low-protein diets in diabetic chronic kidney disease (ckd) patients: Are they feasible and worth the effort?. Nutrients.

[20] Yen C.L., Tu K.H., Lin M.S., Chang S.W., Fan P.C., Hsiao C.C., Chen C.Y., Hsu H.H., Tian Y.C., Chang C.H. (2018). Does a supplemental low-protein diet decrease mortality and adverse events after commencing dialysis? A nationwide cohort study. Nutrients.

[21] Piccoli G.B., Motta D., Martina G., Consiglio V., Gai M., Mezza E., Maddalena E., Burdese M., Colla L., Tattoli F. (2004). Low-protein vegetarian diet with alpha-chetoanalogues prior to pre-emptive pancreas-kidney transplantation. Rev. Diabet. Stud..

[22] Wang J.H., Skeans M.A., Israni A.K. (2016). Current status of kidney transplant outcomes: Dying to survive. Adv. Chronic. Kidney Dis..

[23] Akkina S.K., Connaire J.J., Snyder J.J., Matas A.J., Kasiske B.L. (2008). Earlier is not necessarily better in preemptive kidney transplantation. Am. J. Transplant..

[24] Bohlke M. (2012). Dialysis and kidney transplantation: Why have our rehabilitation hopes not been achieved fully?. Am. J. Kidney Dis..

[25] Jansz T.T., Bonenkamp A.A., Boereboom F.T.J., van Reekum F.E., Verhaar M.C., van Jaarsveld B.C. (2018). Health-related quality of life compared between kidney transplantation and nocturnal hemodialysis. PLoS ONE.

[26] Vollmer W.M., Wahl P.W., Blagg C.R. (1983). Survival with dialysis and transplantation in patients with end-stage renal disease. N. Engl. J. Med..

[27] Hsing A.W., Ioannidis J.P. (2015). Nationwide population science: Lessons from the taiwan national health insurance research database. JAMA Intern. Med..

[28] Lin L.Y., Warren-Gash C., Smeeth L., Chen P.C. (2018). Data resource profile: The national health insurance research database (nhird). Epidemiol. Health.

[29] Hsieh C.Y., Su C.C., Shao S.C., Sung S.F., Lin S.J., Kao Yang Y.H., Lai E.C. (2019). Taiwan’s national health insurance research database: Past and future. Clin. Epidemiol..

[30] Hsieh M.S., Chiu C.S., How C.K., Chiang J.H., Sheu M.L., Chen W.C., Lin H.J., Hsieh V.C., Hu S.Y. (2016). Contrast medium exposure during computed tomography and risk of development of end-stage renal disease in patients with chronic kidney disease: A nationwide population-based, propensity score-matched, longitudinal follow-up study. Medicine.

[31] Lin C.C., Wu Y.T., Yang W.C., Tsai M.J., Liu J.S., Yang C.Y., Li S.Y., Ou S.M., Tarng D.C., Hsu C.C. (2017). Angiotensin receptor blockers are associated with lower mortality than ace inhibitors in predialytic stage 5 chronic kidney disease: A nationwide study of therapy with renin-angiotensin system blockade. PLoS ONE.

[32] Wu C.S., Lai M.S., Gau S.S., Wang S.C., Tsai H.J. (2014). Concordance between patient self-reports and claims data on clinical diagnoses, medication use, and health system utilization in taiwan. PLoS ONE.

[33] Hsieh C.Y., Chen C.H., Li C.Y., Lai M.L. (2015). Validating the diagnosis of acute ischemic stroke in a national health insurance claims database. J. Formos. Med. Assoc..

[34] Elze M.C., Gregson J., Baber U., Williamson E., Sartori S., Mehran R., Nichols M., Stone G.W., Pocock S.J. (2017). Comparison of propensity score methods and covariate adjustment: Evaluation in 4 cardiovascular studies. J. Am. Coll. Cardiol..

[35] Ikizler T.A., Burrowes J.D., Byham-Gray L.D., Campbell K.L., Carrero J.J., Chan W., Fouque D., Friedman A.N., Ghaddar S., Goldstein-Fuchs D.J. (2020). Kdoqi clinical practice guideline for nutrition in ckd: 2020 update. Am. J. Kidney Dis..

[36] Orozco-Guillien A.O., Munoz-Manrique C., Reyes-Lopez M.A., Perichat-Perera O., Miranda-Araujo O., D’Alessandro C., Piccoli G.B. (2021). Quality or quantity of proteins in the diet for ckd patients: Does "junk food" make a difference? Lessons from a high-risk pregnancy. Kidney Blood Press Res..

[37] Bastani B. (2019). The present and future of transplant organ shortage: Some potential remedies. J. Nephrol..

[38] Taylor D., Robb M., Casula A., Caskey F. (2017). Uk renal registry 19th annual report: Chapter 11 centre variation in access to kidney transplantation (2010–2015). Nephron.

[39] Chung C.M., Lin M.S., Hsu J.T., Hsiao J.F., Chang S.T., Pan K.L., Lin C.L., Lin Y.S. (2017). Effects of statin therapy on cerebrovascular and renal outcomes in patients with predialysis advanced chronic kidney disease and dyslipidemia. J. Clin. Lipidol..

[40] Cupisti A., Brunori G., Di Iorio B.R., D’Alessandro C., Pasticci F., Cosola C., Bellizzi V., Bolasco P., Capitanini A., Fantuzzi A.L. (2018). Nutritional treatment of advanced ckd: Twenty consensus statements. J. Nephrol..

[41] Piccoli G.B., Capizzi I., Vigotti F.N., Leone F., D’Alessandro C., Giuffrida D., Nazha M., Roggero S., Colombi N., Mauro G. (2016). Low protein diets in patients with chronic kidney disease: A bridge between mainstream and complementary-alternative medicines?. BMC Nephrol..

