# Supplementary material for: Supplemented Low-Protein Diet May Delay the Need for Preemptive Kidney Transplantation: A Nationwide Population-Based Cohort Study
Source: Nutrients. 2021 Aug 28;13(9):3002. doi: 10.3390/nu13093002 (PMC8467708; doi:10.3390/nu13093002)
Supplement: Supplementary file 1 [file nutrients-13-03002-s001.zip › nutrients-1340668-supplementary.pdf]

Supplemental Table S1. ICD diagnostic codes

Variable	ICD-9 CM code	ICD-10 CM code
Dialysis	585.xx with dialysis treatment	N18 with dialysis treatment
Interstitial nephritis	583.89	N11.8, N11.9, N14, N15.8 , N15.9
Obstructive nephropathy	599.6	N13 with N18.3 to N18.6 at the same time
Polycystic kidney	753.12, 753.13, 753.14	Q61.1, Q61.2, Q61.3
Hypertensive kidney disease	403.90	I12
Diabetic nephropathy	583.81	E08.2, E09.2, E10.2, E11.2, E13.2
Chronic glomerulonephritis	581.xx, 582.xx	N03, N04
Hypertension	401.xx-405.xx with any anti-hypertension drugs	I10-I15, N262 with any anti-hypertension drugs
Diabetes mellitus	250.xx with any oral hypoglycemic drugs and insulin	E08-E13 with any oral hypoglycemic drugs and insulin
Dyslipidemia	272.0-272.4 with any anti-hyperlipidemia drugs	E77, E780, E781, E782, E783, E784, E785, E786, E881, E753, E755, E882, E756, E789, E7521, E7522, E7524, E7130, E7879, E7881, E7889, E8889, E7870 with any anti-hyperlipidemia drugs
Gouty arthritis	274.xx	M10, M1A.0, M1A.2, M1A.3, M1A.4, M1A.9, N20.0
Peptic ulcer / GERD	531.xx-534.xx	K25–K28
Ischemic heart disease	410.xx-414.xx	I20-I24
Liver cirrhosis	571.2, 571.5, 571.6	K702-K703, K741-K746
Heart failure	428.xx	I50
Ischemic stroke	433.xx-437.xx	I66, I65.1, I65.0, I65.8, I65.9, I63.6, I63.8,

Variable	ICD-9 CM code	ICD-10 CM code
		I63.9, G45.0, G45.8, G45.1, G45.2, G46.0, G46.1, G46.2, G45.9, G45.4, G46.3, G46.4, G46.5, G46.6, G46.7, G46.8, I67.0, I67.1, I67.2, I67.4, I67.5, I67.6, I67.7, I67.9, I68.0, I68.2, I68.8
Hemorrhage stroke	430.xx-432.xx	I60-I62
Myocardial infarction	410.xx, 412.xx	I21-I22
Acute myocardial infarction	410.xx	I21-I22
Cardiovascular death	390.xx – 459.xx, 785.5x	I00-I99, R570, R579
Infection death	559, 0031, 0362, 0380, 03810, 03811, 03812, 03819, 0382, 0383, 03840, 03841, 03842, 03843, 03844, 03849, 0388, 0389, 04082, 0545, 1125, 78552, 7907, 7908, 99591, 99592, 03282, 03640, 03641, 03642, 03643, 07420, 07421, 07422, 07423, 11281, 11503, 11504, 11593, 11594, 1303, 3910, 3911, 3912, 3918, 3919, 3920, 4210, 4211, 4219, 4220, 42292, 00321, 0360, 0361, 0470, 0471, 0478, 0479, 048, 0490, 0491, 0498, 0499, 0530, 05310, 05314, 0543, 05472, 05474, 0550, 05600, 05601, 05609, 05821, 05829, 0621, 0622, 0623, 0625, 0628, 0629, 0638, 0639, 064, 06641, 06642, 0721, 0722, 11283, 1142, 11501, 11591, 1300, 3200, 3201, 3202, 3203, 3207, 32081, 32082, 32089,	B05.9, A02.1, A39.2, H61.001, A41.01, H65.30, A41.1, A40.3, A41.4, A41.3, A41.51, A41.52, A41.53, A41.59, A41.89, A41.9, B00.7, A15.0, R78.81, B34.9, A36.81, A39.53, A39.51, A39.52, B33.23, B33.21, B33.22, B37.6, B39.4, B39.4, B39.9, B39.9, A17.0, I01.1, I01.2, I01.8, I01.9, I39, I33.9, I40.0, A02.21, H44.001, A39.81, J34.2, A87.0, A87.0, A87.9, A03.8, J40, A87.1, A85.0, A86, K22.0, K25.4, B00.4, B00.3, K40.40, B06.01, B06.02, N03.1, N03.9, A83.1, A83.2, A83.3, A83.5, A83.6, A83.9, A84.8, A84.9, A06.5, O70.1, O70.2, B26.1, B26.2, B37.5, A15.0, B39.4, B39.9, G00.1, G00.2, G00.3, G01, G00.8, G00.9,

Variable	ICD-9 CM code	ICD-10 CM code
	3209, 3210, 3211, 3212, 3230, 32301, 32302, 3231, 3234, 32341, 32342, 3240, 3241, 3249, 03283, 5670, 5671, 5672, 56721, 56722, 56723, 56729, 56789, 5679, 0030, 0038, 0039, 0040, 0041, 0043, 0048, 0049, 0050, 0051, 0052, 0053, 0054, 00581, 00589, 0059, 0071, 0074, 0075, 00800, 00801, 00802, 00803, 00804, 00809, 0081, 0082, 0083, 00841, 00842, 00843, 00844, 00845, 00846, 00847, 00849, 0085, 00861, 00862, 00863, 00864, 00865, 00866, 00867, 00869, 0088, 0090, 0091, 0092, 0093, 0392, 0700, 0701, 07043, 07053, 0723, 07271, 11285, 1305, 5400, 5401, 5409, 541, 542, 56201, 56203, 56211, 56213, 566, 56781, 5695, 5720, 5721, 5750, 57510, 03284, 0720, 59010, 59011, 5902, 5903, 59080, 59081, 5909, 5950, 5954, 59589, 5959, 5970, 59800, 59801, 5990, 6010, 6012, 6013, 6014, 6019, 6031, 6040, 60490, 60491, 6071, 6072, 6080, 6084, 6140, 6142, 6143, 6145, 6150, 6159, 6163, 6164, 00322, 01100, 01101, 01102, 01103, 01104, 01105, 01106, 01110, 01111, 01112, 01113, 01114, 01115, 01116, 01120, 01121, 01122, 01123, 01124, 01125, 01126, 01130, 01131,	G00.8, G00.9, G02, G02, G05.3, G05.3, G06.1, G06.2, A36.89, B06.82, K65.0, B06.81, A30.5, A02.8, A02.9, A48.0, A03.1, A03.3, A03.8, A03.9, B03, A05.1, A05.2, A05.8, A05.3, A05.5, A05.4, A05.9, A07.1, A07.2, A07.4, S02.0XXA, A04.0, A04.1, A04.2, A04.3, A04.4, A04.8, A04.8, A04.8, A04.8, A04.8, A04.5, A04.6, A04.7, A04.8, A04.8, A04.8, A04.9, A08.0, A08.2, A08.11, A08.19, A08.31, A08.32, A08.39, A08.39, A08.4, A50.01, A09, A09, A09, A42.1, L84, B15.9, B17.2, B17.2, B26.3, B26.81, B37.82, A17.0, K35.3, K35.80, A60.00, B00.2, K57.00, K57.01, K57.20, K57.21, K61.0, K63.0, K75.1, A36.85, M08.1, N10, N15.1, N28.84, N16, N15.9, N30.80, N30.80, N30.90, N37, N41.2, N41.3, N51, N41.9, N43.1, N51, N47.6, N48.21, N49.1, N70.91, N73.0, N73.3, N71.9, N75.1, N76.4, A02.22, A15.0, A15.0, A15.0, A15.0, A15.0, A15.0, A15.0, A15.0, A15.0, A15.0, A15.0, A15.0, A15.0, A15.0, A15.0, A15.0, A15.0, A15.0, A15.5, A15.5, A15.5, A15.5, A15.5, A15.5, A15.0, A15.0, A15.0, A15.0, A15.0, A15.0,

Variable	ICD-9 CM code	ICD-10 CM code
	01132, 01133, 01134, 01135, 01136, 01150, 01151, 01152, 01153, 01154, 01155, 01156, 01160, 01161, 01162, 01163, 01164, 01165, 01166, 01170, 01171, 01172, 01173, 01174, 01175, 01176, 01180, 01181, 01182, 01183, 01184, 01185, 01186, 01190, 01191, 01192, 01193, 01194, 01195, 01196, 0310, 0330, 0338, 0339, 0391, 0521, 0551, 0730, 0796, 1124, 1140, 1145, 11505, 11595, 1304, 1363, 4650, 4658, 4659, 4660, 46611, 46619, 4800, 4801, 4802, 4803, 4808, 4809, 481, 4820, 4821, 4822, 48230, 48231, 48232, 48239, 48240, 48241, 48242, 48249, 48281, 48282, 48283, 48284, 48289, 4829, 4830, 4831, 4838, 4841, 4843, 4846, 4847, 4848, 485, 486, 4870, 4871, 488, 4880, 4881, 490, 49122, 4941, 5100, 5109, 5111, 5130, 5131, 5192, 0311, 03285, 0390, 0400, 37601, 6800, 6801, 6802, 6803, 6804, 6805, 6806, 6807, 6808, 6809, 68100, 68101, 68110, 68111, 6819, 6820, 6821, 6822, 6823, 6824, 6825, 6826, 6827, 6828, 6829, 684, 6850, 6868, 6869, 72886, 9101, 9103, 9109, 9111, 9113, 9119, 9121, 9123, 9129, 9131, 9133, 9139, 9141, 9143, 9149, 9151, 9153, 9159,	A15.0, F07.0, E75.23, A37.80, A37.90, A42.0, B01.2, B05.2, M86.00, B97.4, A15.0, A15.0, B39.0, B39.9, A17.0, A17.82, J06.9, J06.9, J21.0, J21.1, J12.1, J12.2, J12.3, J12.9, J13, J15.1, J14, J15.4, J15.3, J15.4, J15.211, J15.29, J15.8, J15.5, J15.6, A48.1, J15.8, J15.9, J16.0, J16.8, B25.0, A37.91, B44.0, J17, J17, J18.0, J18.8, J09.X2, J40, J47.0, J86.9, J90, J85.3, J98.5, A31.1, A36.3, I00, H05.011, L02.12, L02.221, L02.421, L02.521, L02.32, L02.425, L02.621, L02.821, L02.92, L03.011, L03.031, L03.019, L02.11, L02.211, L02.411, L02.511, L02.31, L02.415, L02.611, L02.811, L02.91, L01.00, B78.1, L08.9, M72.6, L08.89, L08.89, L08.89, L08.89, L08.89, L08.89, L08.89, L08.89, L08.89, L08.89, L08.89, L08.89, L08.89, L08.89, L08.89, A51.43, L08.89, L08.89, A51.46, L08.89, A51.49, L08.89, L08.89, L08.89,

Variable	ICD-9 CM code	ICD-10 CM code
	9161, 9163, 9169, 9171, 9173, 9179, 9191, 9193, 9199, 00323, 00324, 03682, 37603, 05671, 71100, 71101, 71102, 71103, 71104, 71105, 71106, 71107, 71108, 71109, 71140, 71141, 71142, 71143, 71144, 71145, 71146, 71147, 71148, 71149, 71150, 71151, 71152, 71153, 71154, 71155, 71156, 71157, 71158, 71159, 71160, 71161, 71162, 71163, 71164, 71165, 71166, 71167, 71168, 71169, 71180, 71181, 71182, 71183, 71184, 71185, 71186, 71187, 71188, 71189, 71190, 71191, 71192, 71193, 71194, 71195, 71196, 71197, 71198, 71199, 73000, 73001, 73002, 73003, 73004, 73005, 73006, 73007, 73008, 73009, 73020, 73021, 73022, 73023, 73024, 73025, 73026, 73027, 73028, 73029, 73080, 73081, 73082, 73083, 73084, 73085, 73086, 73087, 73088, 73089, 73090, 73091, 73092, 73093, 73094, 73095, 73096, 73097, 73098, 73099, 99662, 99931, 99668, 53086, 53641, 56961, 99660, 99661, 99663, 99664, 99665, 99666, 99667, 99669, 99731, 99802, 99851, 99859, 9993, 99939	L08.89, L08.89, L08.89, A02.23, A02.24, A39.83, H05.021, B06.82, M00.011, M00.021, M00.031, M00.041, M00.051, M00.061, M00.071, M00.08, M00.09, M01.X11, M01.X21, M01.X31, M01.X41, M01.X51, M01.X61, M01.X71, M01.X8, M01.X9, M01.X11, M01.X21, M01.X31, M01.X41, M01.X51, M01.X61, M01.X71, M01.X8, M01.X9, M01.X11, M01.X21, M01.X31, M01.X41, M01.X51, M01.X61, M01.X71, M01.X8, M01.X9, M01.X11, M01.X21, M01.X31, M01.X41, M01.X51, M01.X61, M01.X71, M01.X8, M01.X9, M01.X19, M01.X29, M01.X39, M01.X49, M01.X59, M01.X69, M01.X79, M01.X8, M01.X9, M86.011, M86.021, M86.031, M86.041, M86.051, M86.061, M86.071, M86.08, M86.09, M86.9, M86.9, M86.9, M86.9, M86.9, M86.9, M86.9, M46.20, M86.9, M90.811, M90.821, M90.831, M90.841, M90.851, M90.861, M90.871, M90.88, M90.89, M86.9, M86.9, M86.9, M86.9, M86.9, M86.9, M86.9, M46.30, M86.9, T80.211A, T85.71XA, K94.22,

Variable	ICD-9 CM code	ICD-10 CM code
		K94.02, T82.6XXA, T85.79XA, T83.51XA, T83.59XA, T84.50XA, T84.60XA, T85.72XA, T81.4XXA, K68.11, N98.0
Sepsis	038.xx, 790.7	A40, A41
Malignancy	140.xx-208.xx	C00-C96
Osteoporosis-related fracture	733.1x, 805.xx-829.xx, excluding E810.x-E819.x	S02, S12, S22, S32, S42, S52, S62, S72, S82, S93, M80 and without accident mark

ICD, International Classification of Diseases; CM, Clinical Modification.
